# A Spot Reminder System for the Visually Impaired Based on a Smartphone Camera

**DOI:** 10.3390/s17020291

**Published:** 2017-02-04

**Authors:** Hotaka Takizawa, Kazunori Orita, Mayumi Aoyagi, Nobuo Ezaki, Shinji Mizuno

**Affiliations:** 1 University of Tsukuba, 1-1-1 Tennodai, Tsukuba 305-8573, Ibaraki, Japan; kc_orita@pr.cs.tsukuba.ac.jp; 2 Aichi University of Education, 1 Hirosawa, Igaya, Kariya 448-8542, Aichi, Japan; maoyagi@auecc.aichi-edu.ac.jp; 3 National Institute of Technology, Toba College, 1-1 Ikegami, Toba 517-8501, Mie, Japan; ezaki@toba-cmt.ac.jp; 4 Aichi Institute of Technology, 1247 Yachigusa, Yakusa, Toyota 470-0392, Aichi, Japan; s_mizuno@aitech.ac.jp

**Keywords:** spot reminder, visually impaired individuals, smartphone cameras, scale invariant feature transform, image matching, user study

## Abstract

The present paper proposes a smartphone-camera-based system to assist visually impaired users in recalling their memories related to important locations, called spots, that they visited. The memories are recorded as voice memos, which can be played back when the users return to the spots. Spot-to-spot correspondence is determined by image matching based on the scale invariant feature transform. The main contribution of the proposed system is to allow visually impaired users to associate arbitrary voice memos with arbitrary spots. The users do not need any special devices or systems except smartphones and do not need to remember the spots where the voice memos were recorded. In addition, the proposed system can identify spots in environments that are inaccessible to the global positioning system. The proposed system has been evaluated by two experiments: image matching tests and a user study. The experimental results suggested the effectiveness of the system to help visually impaired individuals, including blind individuals, recall information about regularly-visited spots.

## 1. Introduction

**Figure 1:**
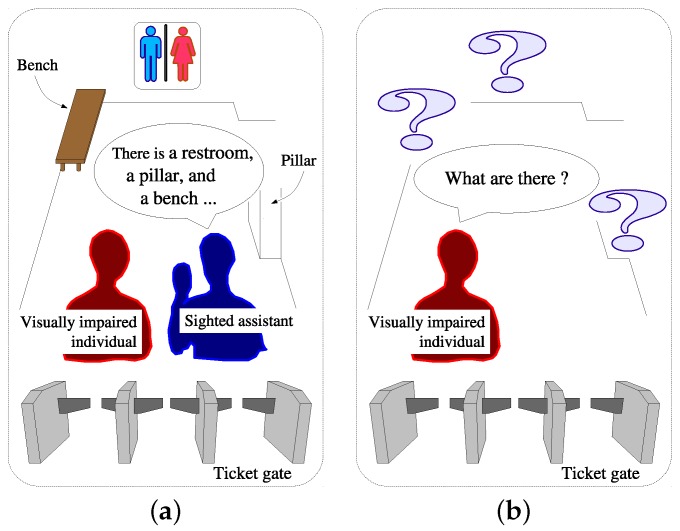
(**a**) One day; (**b**) Several days later. One day, a visually impaired individual visited a ticket gate in a station with a sighted assistant. The assistant taught the individual that there was a restroom in the front, a pillar on the right, and a bench on the left. Several days later, the individual returned alone, but forgot about the restroom and the bench. Therefore, the individual was not able to use them.

In 2014, the World Health Organization estimated the number of visually impaired individuals to be at approximately 285 million worldwide [1]. Many are trained by sighted assistants to move along their daily routes, for example, from home to the office. During such training, they are often taught information about important locations, called *spots*, along the routes. If they remember the information, their quality of life (QOL) would be maintained. Otherwise, they would be forced to suffer inconvenience. Figure 1 illustrates a typical situation in which the QOL of a visually impaired individual is strongly affected by whether the individual remembers the information about a spot. It is necessary to build an assistive system to help a visually impaired user recall information about environments.

A number of research groups have proposed obstacle detection systems to notify visually impaired users about the positions of obstacles which were detected using laser sensors [2,3,4,5,6,7,8,9,10], ultrasonic sensors [11,12,13,14,15,16,17,18,19,20,21,22] single charge-coupled devices (CCD) cameras [23,24,25,26,27], stereoscopic cameras [28,29,30,31,32,33,34,35,36,37,38], or RGB-D cameras [39,40,41,42,43]. These systems allow users to walk safely while avoiding obstacles (such as the pillar in Figure 1), even if they forget or do not know the positions of the obstacles.

The systems can warn users of obstacles in their vicinity but cannot tell the users what the objects are.

Other research groups have proposed assistive systems to recognize objects such as drug packages [44], podiums [45], classroom doors [45,46,47,48], and pathways [49,50], using barcodes [44,51], radio frequency identification tags [22,46,52,53], Bluetooth devices [54], augmented reality markers [45,47,48], circular markers [49], wireless network devices [50], or visible light communication devices [55,56]. These physical devices can help visually impaired users identify the objects, but in practice it is difficult to deploy them in an everyday environment.

Sensor-based systems have been developed to recognize color blocks [57], benches [58], tables [59], staircases [60,61,62,63], and elevators [64], using laser range sensors [62,63], laser pointers combined with a CCD camera [61], or Kinect sensors [57,58,59,64]. These systems allow visually impaired users to find and use target objects. For example, in Figure 1b, the individual can find the bench to take a rest. These systems can obtain information from the environment but cannot add information to the environment.

Smartphone-based systems have been proposed to navigate visually impaired individuals in indoor and outdoor environments. Elloumi et al. developed an algorithm for indoor pedestrian localization based on a smartphone camera fixed on a body harness [65]. Götzelmann et al. introduced an approach to combine a physical tactile map with an interactive application running on a smartphone for outdoor navigation [66]. These systems are also incapable of appending information to the environment.

Social platform systems have been proposed as a way of sharing barrier-free information among people with and without disabilities [67,68]. Anyone can upload information to maps on websites managed by the systems, and anyone can download the information from the websites. However, these systems require visually impaired users to search through a large amount of information uploaded by other people and to verify the downloaded information. Sekai Camera was a social platform system that was able to attach virtual information to the real world and display it on the screen of a smartphone. The Sekai Camera system had the same problem as the above platform systems and in addition was mainly designed for sighted people (The services of Sekai Camera were terminated in 2014.).

In this paper, we propose a smartphone-camera-based reminder system to help visually impaired users recall their own memories of spots that they visited. The memories are recorded as voice memos, which can be played back when the users return to the same spots. Spot-to-spot correspondence is determined by image matching between scene images obtained by the smartphone camera at the spots. The proposed system was implemented as an application on an Android smartphone and evaluated using two experiments: image matching tests and a user study.

This paper is organized as follows: Section 2 describes the outline of the spot reminder system, Section 3 explains the system implementation, Section 4 shows the experimental results, Section 5 discusses these results, and Section 6 concludes the paper.

## 2. Outline of the Spot Reminder System

**Figure 2:**
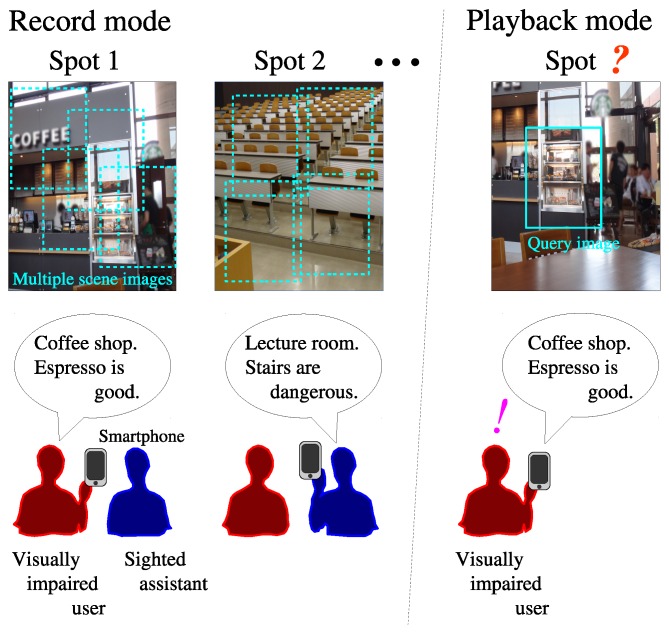
Outline of the spot reminder system.

Figure 2 illustrates the outline of the spot reminder system in two modes: record and playback.

In the record mode, at each spot, a visually impaired user or a sighted assistant takes multiple scene images (depicted by frames with dotted lines in Figure 2) using a smartphone camera and then records a voice memo about the spot (for example, “Coffee shop. Espresso is good.”) on the smartphone. From the multiple images, feature points called *keypoints* are extracted using the Scale Invariant Feature Transform (SIFT) [69,70]. The images are then merged into one panoramic image using the SIFT-based image stitching technique [70,71]. The panoramic image and the voice memo are stored in a dictionary on the smartphone.

In the playback mode, when the user returns to one of the recorded spots, he or she takes a scene image for a search query (a frame with solid lines). SIFT-based image matching is performed between the query image and the dictionary images, and then the current spot is identified from the matching result. The smartphone plays the associated voice memo, which can help the user recall his or her memory related to the spot.

**Figure 3:**
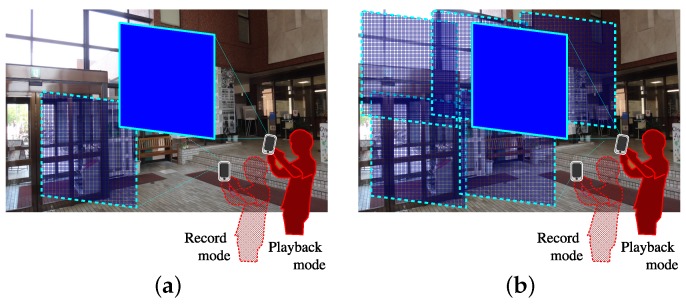
(**a**) Single scene image; (**b**) Panoramic scene image. If a single scene image is used for image matching, the system would fail to identify the current spot. By using a panoramic image, the current spot can be identified correctly.

Figure 3 explains the reason why panoramic scene images are used in the proposed system. In Figure 3a, a visually impaired user attempts to identify the current spot on the basis of image matching between a single scene image taken in the record mode and a query image taken in the playback mode. These images are depicted by frames with dotted and solid lines, respectively. It is difficult for the visually impaired user to set a camera direction in the playback mode so that the frames overlap sufficiently, and therefore it is likely that the image matching would fail. Our previous navigation system [72] and the VizMap localization system, proposed by Gleason et al. [73], used single scene images for spot identification and therefore had the same difficulty in image matching. In contrast, if a panoramic image is produced from multiple scene images with the help of a sighted assistant and is used for the image matching, it would be more promising as shown in Figure 3b. Image matching techniques were also used to recognize objects such as food packages in [74], where visually impaired users were required to set cameras toward target objects precisely. In our method, visually impaired users are less demanding.

### 2.1. Keypoint Extraction by SIFT

SIFT extracts keypoints through a detection phase followed by a description phase.

In the detection phase, a scene image is first enhanced by histogram equalization [75], which can increase the number of reliable keypoints [76]. The enhanced image is smoothed by the Gaussian filters of various scales (i.e., variances). By subtracting the adjacent Gaussian-smoothed images, Difference-of-Gaussian (DoG) images are produced. From the sequential DoG images, local extremum pixels are detected as keypoint candidates. Their sub-pixel positions and scales are obtained by interpolation based on the quadratic Taylor expansion of the DoG functions. By eliminating the low-contrast or on-the-edge candidates, the final keypoints are selected.

In the description phase, gradient magnitudes and orientations are computed for pixels in the Gaussian-smoothed images, and then the orientations of the keypoints are calculated from the 36-bin histograms of the gradient orientations weighted by their magnitudes. A squared region of interest (ROI) is set at each keypoint. Its size and orientation are determined from the scale and orientation of the keypoint, respectively. The ROI is divided into 4×4 blocks, and for each block, the 8-bin histogram of the gradient orientations weighted by the magnitudes is calculated. From the 4×4×8 elements, a 128-dimensional feature vector is produced as the descriptor of the keypoint. The 128-dimensional feature vector is reasonably invariant against changes in scaling, rotation and illumination of images.

### 2.2. SIFT-Based Image Stitching in the Record Mode

Multiple scene images taken at each spot are sorted according to their timestamps, and are represented as $I_{n}$ (n=1,⋯,N). The first and second images, $I_{1}$ and $I_{2}$, are defined as reference and floating images, $I^{r}$ and $I^{f}$, respectively. The following algorithm is iterated until all the images are processed.

1. From $I^{r}$ and $I^{f}$, keypoints are extracted. They are represented as $k_{i}^{r}$ (i=1,⋯,I) and $k_{j}^{f}$ (j=1,⋯,J), respectively, and their 128-dimensional feature vectors are represented as $v_{i}^{r}$ and $v_{j}^{f}$, respectively. Each keypoint in the reference image is paired with the most similar keypoint in the floating image. The similarity is evaluated by the following Euclidian distance between the 128-dimensional feature vectors of the keypoints:
  (1) $$d\left(i,j\right)={\left|v_{i}^{r}-v_{j}^{f}\right|}^{2}.$$
  The keypoint pairs are represented as $p_{k}$ (k=1,⋯,K).
2. Some of the keypoint pairs come from the same objects observed in the reference and floating images. However, other pairs come from different objects and would cause errors in image stitching. They are removed by using the random sample consensus algorithm [77] as follows:
  (a) Four keypoint pairs, $p_{k_{1}}$, $p_{k_{2}}$, $p_{k_{3}}$ and $p_{k_{4}}$, are chosen randomly.
  (b) A homography matrix [78], *H*, is calculated by applying the direct linear transformation algorithm [79] to the four keypoint pairs.
  (c) For the keypoint pair $p_{k}$, a back projection error, $err(p_{k};H)$, is calculated. If $err\left(p_{k};H\right)<\epsilon_{H}$, $p_{k}$ is determined to be an *inlier*. Otherwise, *outlier*. The inliers and outliers represent the keypoint pairs of the same and different objects, respectively.
  (d) After iterating the above steps from (a) to (c), the algorithm determines the optimal homography matrix that produces the most inliers.
3. The floating image is transformed using the optimal homography matrix and merged into the reference image. The merged image is defined as a new reference image, and the next scene image, $I_{n}$ (n>2), is defined as a new floating image. The algorithm returns to step 1.

The final reference images are stored into the image dictionary on a smartphone.

### 2.3. SIFT-Based Image Matching in the Playback Mode

A query image is represented as $I^{q}$. The first dictionary image is selected as a checking image, $I^{c}$. The image matching is performed by the following algorithm:

1. Keypoints are extracted from $I^{q}$ and $I^{c}$, and keypoint pairs are produced in the same manner as Section 2.2.
2. The geometrical relations between the keypoint pairs are evaluated on the basis of the following six criteria which have been proposed for pedestrian navigation [80,81]:
  (a) too few pairs
  (b) size consistency
  (c) direction consistency
  (d) two-dimensional affine constraint
  (e) area size
  (f) axis inversion
  If all the criteria are satisfied, $I^{c}$ is determined to be matched with $I^{q}$ and the algorithm is terminated.
3. The next dictionary image is selected as $I^{c}$, and the algorithm returns to step 1.

If $I^{q}$ is not matched with any dictionary images, the algorithm determines that there are no match images.

## 3. System Implementation

**Figure 4:**
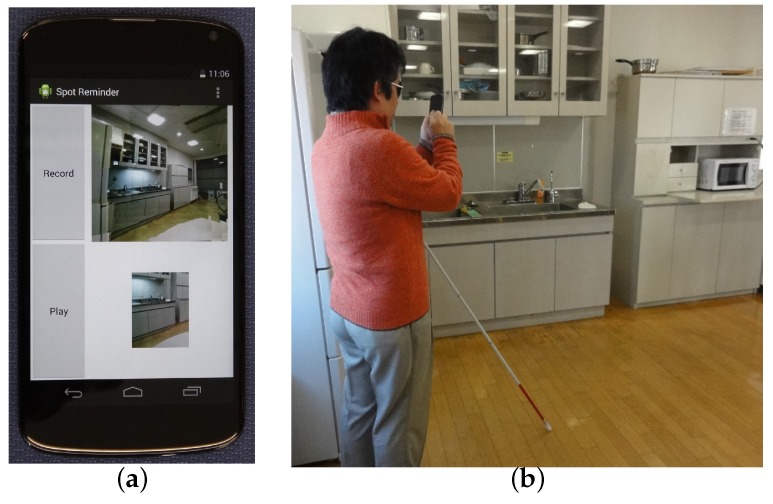
(**a**) Display of the system; (**b**) A user used the system. The spot reminder system.

The spot reminder system was implemented as an application on an Android Google Nexus 4 smartphone as shown in Figure 4a. The application displayed four components on the touch screen. The buttons “Record” and “Play” were able to activate the record and playback modes, respectively. Dictionary and query images allowed a sighted assistant to confirm the images. These images were able to be removed on demand and replaced by the activation buttons displayed full screen.

When a visually impaired user used the spot reminder system, the user stopped walking for safety and set the smartphone as shown in Figure 4b. In the record mode, the user or an assistant pushed the “Record” button to take multiple scene images and input a voice memo to the smartphone. In the playback mode, the user pushed the “Play” button to take a query image and heard the played-back voice memo.

## 4. Experiments

The proposed system was evaluated by image matching tests and a user study. The image matching tests evaluated the accuracy of the image matching between panoramic dictionary images and query images. The user study evaluated the effectiveness of the system in helping participants recall memories related to spots.

The parameters for keypoint extraction, image stitching and image matching were selected experimentally through preliminary experiments.

### 4.1. Image Matching Test

We carried out two types of image matching tests: identification and null response. In the identification test, the system was fed with query images taken at the same spots as dictionary images. The system was required to identify the dictionary images that corresponded to the query images. In the null-response test, the system was fed with query images taken at unknown spots. The system was required to reply that the queries were unknown.

#### 4.1.1. Identification Test

Ten indoor spots and ten outdoor spots were selected for the test. At each spot, one panoramic image was produced, and four single images were taken. The twenty panoramic images were stored as dictionary images in a smartphone, and the eighty single images were input to the smartphone as queries.

Table 1 lists the accuracy of the image matching. *Correct identification* represents a case where the system correctly identified the dictionary image as matching the query image. *Incorrect identification* represents a case where the system mistakenly selected the dictionary image that did not correspond to the query image. *Null response* represents a case where the system determined that the dictionary included no images corresponding to the query image. In the identification test, the null response means a failure of image matching, because the dictionary included at least one image corresponding to each query image.

**Figure 5:**
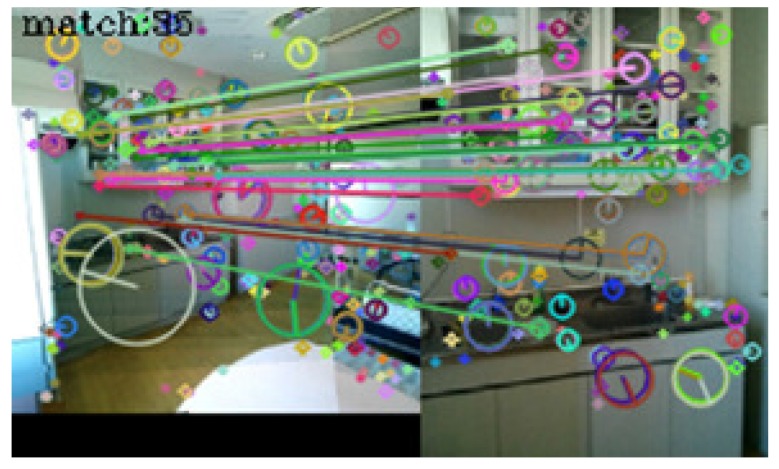
Result of image matching between dictionary and query images taken at the same indoor spot. The images have been resized for better visualization.

**Figure 6:**
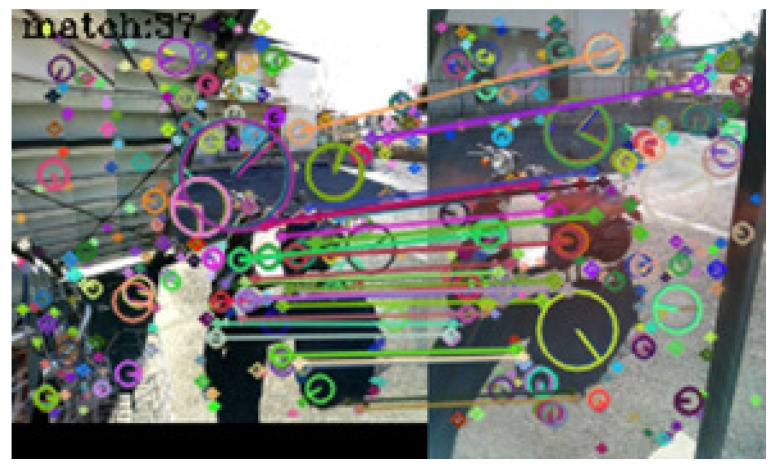
Result of image matching between dictionary and query images taken at the same outdoor spot.

**Figure 7:**
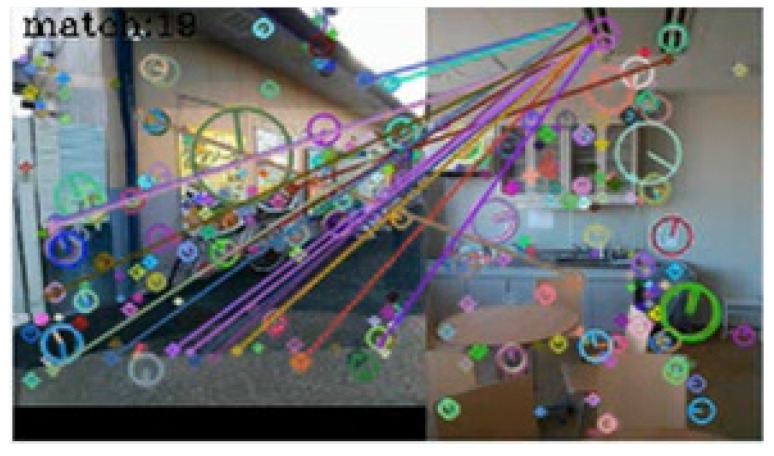
Result of image matching between dictionary and query images taken at different spots.

**Figure 8:**
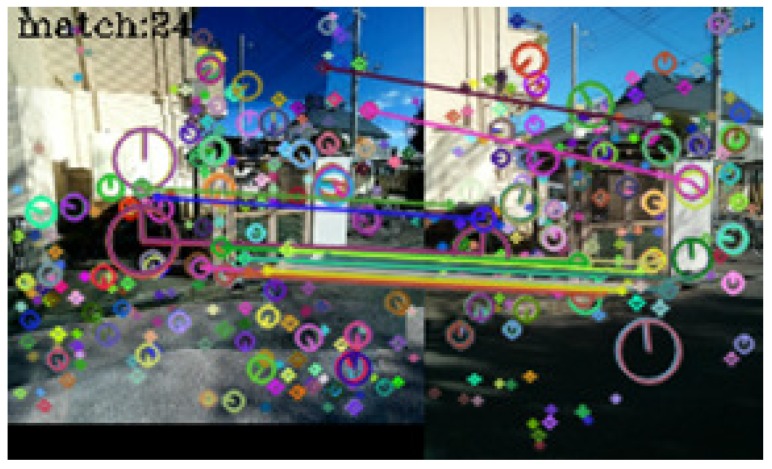
Result of image matching between dictionary and query images taken at the same outdoor spot.

In Table 1, seventy-three query images were identified correctly. No incorrect identifications were made, but in seven cases, the system failed to link the query images to those in the dictionary. Figure 5, Figure 6, Figure 7 and Figure 8 show examples of matching results.

Figure 5 shows the result of image matching between a dictionary image (left) and a query image (right) taken at the same spot in a reinforced concrete building. In the figure, circles represent the scales of keypoints, segments in the circles represent their orientations, and lines between the keypoints represent keypoint pairs. Thirty-six keypoint pairs were obtained from the same objects at the spot. These keypoint pairs satisfied all the criteria of the playback mode, and the system correctly determined that these images were taken at the same spot.

Figure 6 shows the result of image matching of images taken at the same outdoor spot. Thirty-seven keypoint pairs were obtained from the images, and the system identified the spot correctly.

Figure 7 shows the result of image matching at outdoor and indoor spots. Nineteen keypoint pairs were obtained from the images but were generated between different objects. At the playback mode, the direction consistency criterion was not met. Consequently, the system correctly determined that the images were taken from different spots.

Figure 8 shows the result of image matching at the same outdoor spot. Twenty-four keypoint pairs were obtained, but many of them were concentrated in a small region. Consequently, the axis inversion criterion became unstable, and the system failed to identify the spot.

#### 4.1.2. Null-Response Test

Scene images were newly taken at twenty other spots and were input to the system as unknown queries. In the null-response test, null response means that the system succeeded in determining that the spots were unknown. All the query images were correctly determined to be unknown (Table 2).

**Figure 9:**
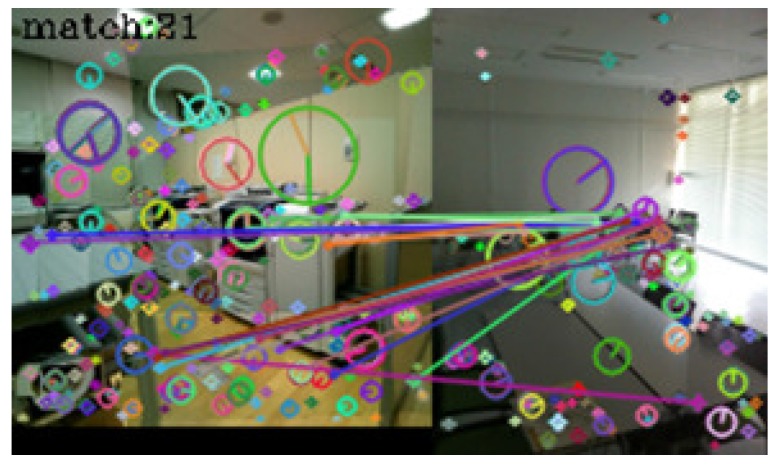
Result of image matching between a dictionary image and an unknown query image.

**Figure 10:**
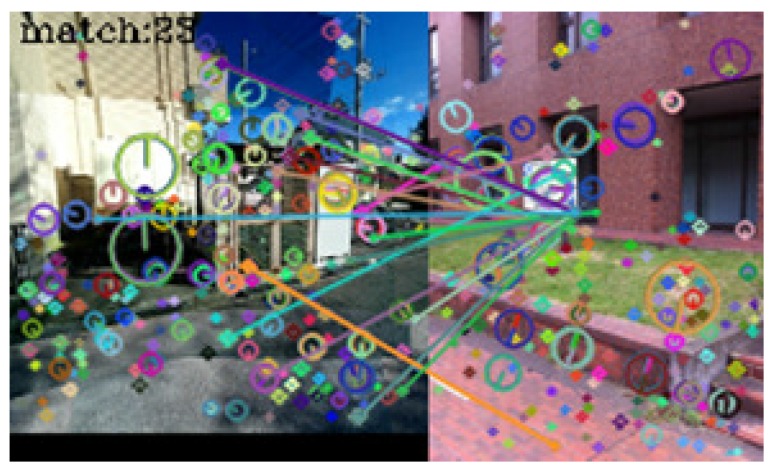
Result of image matching between a dictionary image and an unknown query image.

Figure 9 and Figure 10 show the results of image matching between the dictionary images and the unknown query images. The system successfully determined that they were different spots.

### 4.2. User Study

**Figure 11:**
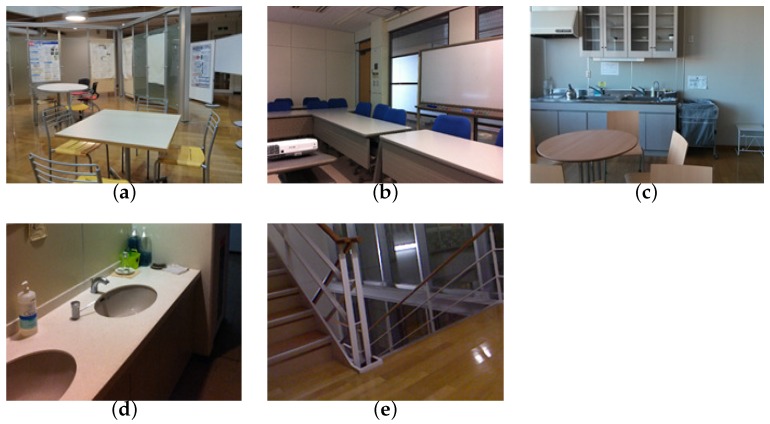
(**a**) Spot 1; (**b**) Spot 2; (**c**) Spot 3; (**d**) Spot 4; (**e**) Spot 5. Five spots used for the user study.

We conducted a user study in which two blindfolded participants, graduate students in their twenties, attempted to recall their memories of five spots in a building located on their university campus (Figure 11).

The participants were given fictitious information about these spots. For example, they were informed that the first spot was a convenience store including a restroom on the right side, an automated teller machine on the left side, a drink section at the right back, and a bread section at the left back. The reason why we used such fictitious information was that the participants were usually living in the building, and therefore they knew the true information about these spots. The fictitious information ensured that the participants did not to use their prior knowledge about these spots.

The user study was conducted as follows:

1. The participants were taken to the spots and given the fictitious information.
2. Ten minutes later, the participants were taken to the same spots again and asked to repeat the information without using the system.
3. The participants were then allowed to use the system and asked to repeat the information again.

Table 3 lists the results of the user study. In this table, “/” and “X” represent the cases where the participants reported the correct and incorrect information, respectively.

The first and second participants made six and two errors, respectively, when not using the proposed system. When using the system, they made no errors.

## 5. Discussion

The main contribution of the proposed system is to enable visually impaired users to associate arbitrary voice memos with arbitrary places and to automatically recover the voice memos when returning to the same places. No special devices or systems are required, except smartphones, and the users do not need to remember the places where the voice memos were recorded. Our preliminary investigation revealed that some visually impaired individuals had used conventional tape/IC recorders to record voice memos. These devices are incapable of linking voice memos with places, requiring the users to search for voice memos corresponding to the current spots. The proposed system, in contrast, can identify the corresponding voice memos automatically, and therefore the users can obtain the necessary information more efficiently.

The advantage of the proposed system over the social platform systems is that visually impaired users can store only the desired information without having to discard irrelevant information uploaded by other people. For example, in the situation shown in Figure 2, the user may be interested only in the espresso coffee in the coffee shop. By excluding irrelevant detail in the record mode, the voice memo will only mention the espresso coffee in the playback mode. In a social platform system, if another user uploaded information about the other food and drink on sale, the user would need to search through the unnecessary information.

Many of the social platform systems [68] used the global positioning system (GPS) to determine the current positions of users. The GPS is unable to operate in many environments, such as the interiors of reinforced concrete buildings. The proposed system uses a vision-based positioning method previously used in other works [81,82]. This method can determine the current positions in locations where GPS is unable to operate.

In the user study, the participants were unable to recall at least 10% of the information about the spots without being prompted by the proposed system. When using the proposed system, they were able to recall all the information correctly. Although the participants were young graduate students and the intervals between the record and playback modes were only ten minutes, the results demonstrated that the proposed system was effective. This could be confirmed by user studies with older participants and longer time intervals.

Image matching methods based on single scene images [83,84] cannot be applied to our problem as described in Section 2. Therefore, we have improved the image matching method by combining with an image stitching method. Stitched images can cover the wider areas of scenes, and therefore visually impaired users can match the scenes even though the directions of smartphone cameras differ somewhat. The accuracy of image matching was not 100% as described in Section 4.1.1, and it would influence the spot identification. The image matching method should be improved. The fourth matching criterion (i.e., two-dimensional affine constraint) used in Section 2.3 is reasonably invariant against the changes of view points. The SIFT can absorb illumination changes to some extent, but cannot if the changes are too large. For example, the system was unable to match a dictionary image taken in sunlight to a query image of the same spot taken at sunset. This can be resolved by collecting scene images under a range of lighting conditions, but this is difficult to do in practice. New techniques, such as Colored SIFT [85], are needed to compensate the large illumination changes.

In the proposed method, visually impaired users should update spot information by themselves. As for the social platform systems, on the other hand, sighted volunteers can update the shared information via the network instead of visually impaired users. In this regard, the social platform systems are superior to the proposed system.

The proposed system can identify spots that a user visited once, but cannot recognize spots where he or she comes for the first time. In addition, if several spots have similar scenes, the system cannot identify the spots. We should improve the identification method and create a new recognition method for more convenience of the visually impaired.

The user study was carried out with a small number of blindfolded young people. With the experimental results, it cannot be said that the proposed system is effective for all visually impaired individuals. However, we think that the results would be able to suggest the effectiveness to people that became sightless lately. Especially, if they have the experiences of using smartphones, they can utilize our system easily because the proposed method is implemented as a typical application on a smartphone. In this paper, we mainly described the proposed system from the viewpoint of system development. In the future, we should evaluate the effectiveness of our system with actual visually impaired individuals.

The calculation time for image matching was less than one second when processing five spots. This would increase as more spots are added to the system. The image matching must be made more efficient to enable the system to deal with a larger number of spots.

## 6. Conclusions

The present paper proposed a spot reminder system to assist visually impaired users in recalling memories related to spots that they visited. The memories were recorded as voice memos, which were able to be played back when the user returned to the spots. The spot-to-spot correspondence was determined by SIFT-based image matching. The system was implemented as an application on an Android smartphone and evaluated by two experiments: image matching tests and a user study. The experimental results suggested the effectiveness of the system to help visually impaired individuals, including blind individuals, recall information about regularly-visited spots.

## Figures and Tables

**Table 1:** Accuracy of the identification test.

	# of Images	Ratio
Correct identification	73	91%
Incorrect identification	0	0%
Null response	7	9%

**Table 2:** Accuracy of the null-response test.

	# of Images	Ratio
Incorrect identification	0	0%
Null response	20	100%

**Table 3:** Results of the user study.

		Participant 1		Participant 2	
		without System	with System	without System	with System
Spot	Information				
1	1	/	/	/	/
	2	/	/	/	/
	3	X	/	/	/
	4	/	/	/	/
2	1	/	/	/	/
	2	/	/	/	/
	3	/	/	/	/
	4	X	/	/	/
3	1	/	/	/	/
	2	/	/	X	/
	3	X	/	/	/
	4	/	/	/	/
4	1	X	/	/	/
	2	X	/	/	/
	3	/	/	/	/
	4	/	/	/	/
5	1	/	/	/	/
	2	X	/	/	/
	3	/	/	/	/
	4	/	/	X	/
Ratio		70%	100%	90%	100%
